# Supplementary material for: Transgene-Free Cynomolgus Monkey iPSCs Generated under Chemically Defined Conditions
Source: Cells. 2024 Mar 21;13(6):558. doi: 10.3390/cells13060558 (PMC10969578; doi:10.3390/cells13060558)
Supplement: Supplementary file 1 [file cells-13-00558-s001.zip › cells-2809253-supplementary.pdf]

Communication

Transgene-free cynomolgus monkey iPSCs generated under chemically defined conditions

Yuliia Tereshchenko [1,2,†], Nesil Esiyok [1,†], Enrique Garea-Rodríguez [3], Daniele Repetto [3], Rüdiger Behr [1,2,*] and Ignacio Rodríguez-Polo [1,2,4,*,†,‡]

1. Supplementary Figures

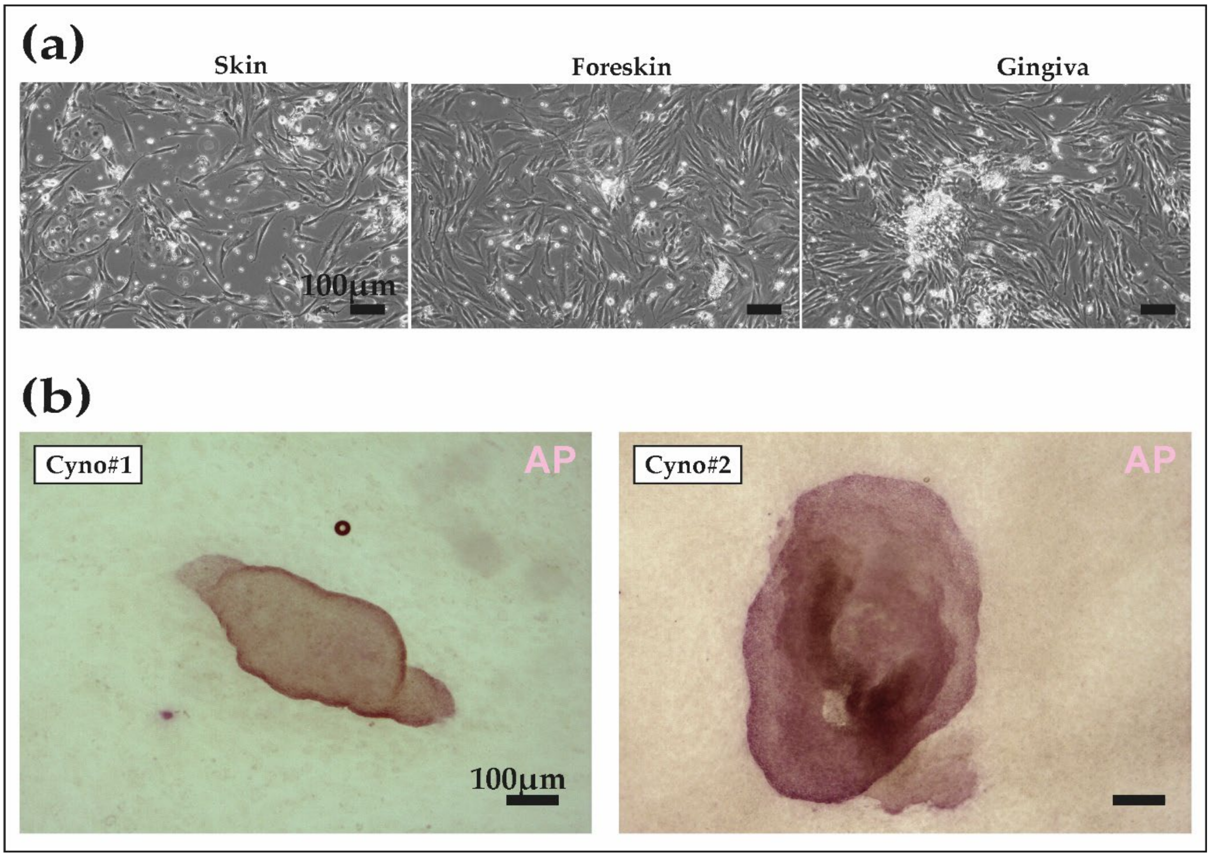

Supplementary Figure S1: Cynomolgus macaque fibroblasts can be reprogrammed using episomal vectors. (**a**) Representative brightfield images of primary fibroblast cell lines derived from skin, gingiva and foreskin biopsies (Cyno#1). Scale bars 100um. (**b**) Putative primary iPSC colonies, after reprogramming, show increased alkaline phosphatase activity in contrast to the surrounded, non-reprogram cells. Scale bars 100um.

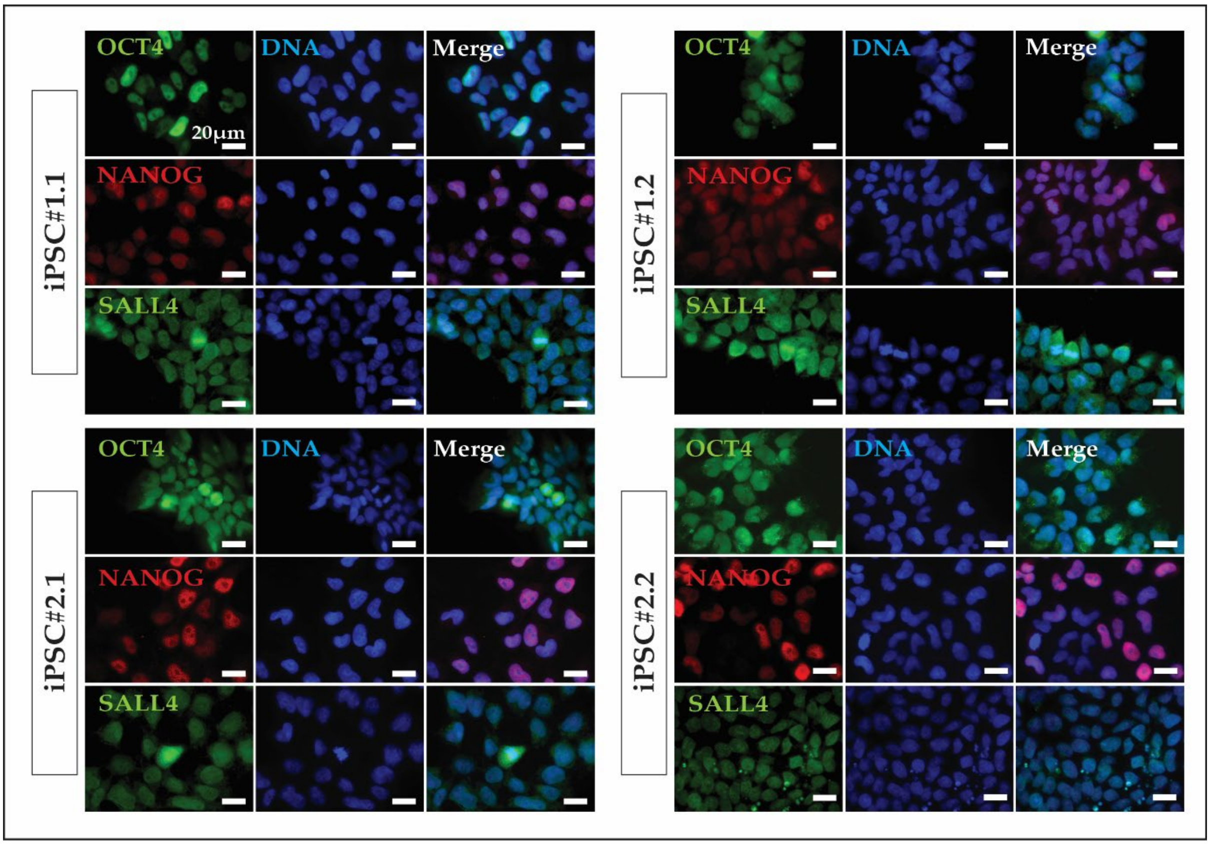

Supplementary Figure S2: Pluripotency marker expression shown by immunofluorescence. The figure includes stainings for the nuclear pluripotency markers depicted in Fig 1, c. Here DNA (DAPI) stainings are also included to demonstrate nuclear localization of the selected epitopes. Scale bars 20µm.

(a)

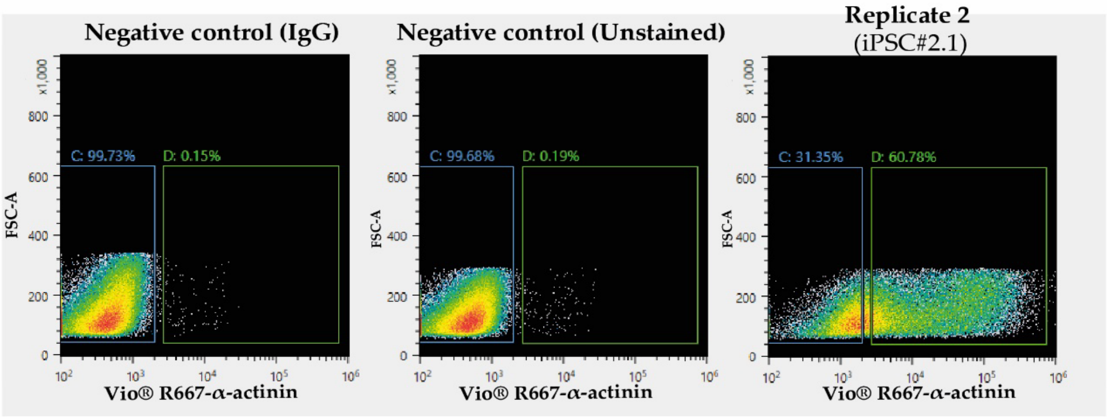

(b)

	Percentage of α-actinin positive cells		
	iPSC#1.1	iPSC#1.2	iPSC#2.1
Replicate 1	8.67	19.66	61.50
Replicate 2	8.45	13.79	60.78
Replicate 3	13.83	17.35	50.36
Replicate 4	5.68	13.2	59.06
Replicate 5			65.12
Replicate 6			69.18
Replicate 7			63.41
Replicate 8			63.81

Supplementary Figure S3: Fluorescence-activated cell sorting (FACS) analysis of macaque iPSC-derived cardiomyocyte population purity before metabolic selection using α-actinin antibody. (a) Gating strategy. (b) Individual values are plotted in Fig., 3 f.
